# The duality principle in the presence of postselection

**DOI:** 10.1038/srep19944

**Published:** 2016-01-29

**Authors:** Jonathan Leach, Eliot Bolduc, Filippo M. Miatto, Kevin Piché, Gerd Leuchs, Robert W. Boyd

**Affiliations:** 1 IPaQS, SUPA, Heriot-Watt, Edinburgh, EH14 4AS, UK; 2 Dept. of Physics, University of Ottawa, 150 Louis Pasteur, Ottawa, Ontario, K1N 6N5 Canada; 3 Max Planck Institute for the Science of Light, Erlangen, Germany; 4 Institute of Optics, University of Rochester, Rochester, USA; 5 SUPA, School of Physics & Astronomy, University of Glasgow, G12 8QQ, UK

## Abstract

The duality principle, a cornerstone of quantum mechanics, limits the coexistence of wave and particle behaviours of quantum systems. This limitation takes a quantitative form when applied to the visibility 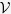 of interference fringes and predictability 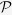 of paths within a two-alternative system, which are bound by the inequality 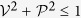. However, if such a system is coupled to its environment, it becomes possible to obtain conditional measures of visibility and predictability, i.e. measures that are conditioned on the state of the environment. We show that in this case, the predictability and visibility values can lead to an apparent violation of the duality principle. We experimentally realize this apparent violation in a controlled manner by enforcing a fair-sampling-like loophole via postselection. This work highlights some of the subtleties that one can encounter while interpreting familiar quantities such as which-alternative information and visibility. While we concentrated on an extreme example, it is of utmost importance to realise that such subtleties might also be present in cases where the results are not obviously violating an algebraic bound, making them harder (but not any less crucial) to detect.

The duality principle provides us with one of the most well-known statements about quantum mechanics: the presence of interference and the existence of which-alternative information are mutually exclusive. For the case of two-dimensional systems (qubits), Greenberger and Yasin defined a quantitative measure of which-alternative information, which we refer to as the predictability 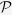, and a quantitative measure of interference, the visibility 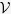
1. They demonstrated the result 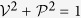 for pure states. They also showed that this formula, in case of a qubit embedded in an environment, generalizes to the duality relation


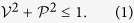


Embedding the qubit in an environment allows us to describe realistic situations. In this case, multiple degrees of freedom can potentially be coupled together. Englert^2^ studied the effect of coupling a qubit to an environment more formally. The deep significance of the duality principle is in the fact that the quantities involved bound each other: the more is known about the alternatives, the less they can interfere and vice versa. This principle has been put to the test, directly and indirectly, many times and in different regimes^3^,^4^,^5^,^6^,^7^,^8^,^9^,^10^. In all of the experimental tests the duality principle prevailed. However, while not in conflict with the duality principle, Menzel *et al.* recently reported high which-alternative information and high-visibility fringes originating from measurements on a single system^11^,^12^; such result falls within the scope of the present work. Following that work, Bolduc *et al.* reported on the apparent violation of the duality relationship from the perspective of fair sampling^13^.

Bergou and Englert have shown various duality relations that apply in the case of a qubit coupled to an arbitrarily large environment^14^. In particular, the most stringent one is


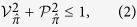


where 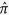 is an observable of the environment. The notation 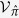 (and similarly for the predictability) should be read as the visibility of the conditional state 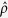, obtained after a measurement of the observable 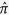. It follows from equation 2 that for any state of the environment the duality principle prohibits simultaneous knowledge of visibility and predictability.

In this work, we experimentally demonstrate a set of conditions in which it is possible to obtain both high-visibility interference fringes and high which-way information. At first this result may seem in conflict with the principles of duality. However, it can be explained in a simple way by noting that in our experiment we purposely fail to satisfy fair sampling. The key feature that makes unfair sampling possible is a non-separable state of the system of interest and its environment. Such correlation allows the measurements of visibility and predictability to be conditioned on successful postselection of different environment outcomes, say 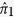 and 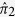, and consequently measure high values for each simultaneously.

The explicit manner in which we control the coupling between different degrees of freedom may seem extreme. However, we note that coupling between degrees of freedom occurs naturally in many physical processes, and it is the key concept for generalised measurements. In addition, postselection of a distribution occurs in many experiments, e.g. when single-mode fibres are used to collect the fundamental mode of a field. The control of coupling and postselection therefore highlights one potential way that can lead to unfair sampling. We find that dramatic results occur for a range of coupling strengths and that even a weak coupling can yield an apparent violation. The physical mechanism behind the coupling can be either explicit, as in the case of this work, or subtle, as in the case of Menzel *et al.*^11^,^12^. Using the simplest possible formalism and experimental implementation of duality, our work raises awareness on one of the potential ways in which the fair-sampling criterion is not satisfied and on the adverse effects that this might have on the interpretation of the experimental results. It is a cautionary tale in the same manner of the work of Romero *et al.* who considered non-locality in the context of high-dimensionally entangled states^15^. In that work, the authors showed that a slight deviation from the ideal measurement settings can inadvertently introduce additional dimensions in the measured state, enabling the apparent measurement of a Bell parameter above the Tsirelson bound. In the same way, we show that a slight coupling between two degrees of freedom can inadvertently produce different states upon postselection, enabling the apparent violation of the duality relationship. We conjecture that the mechanism of coupling and postselection may provide a pathway to seemingly violate also other inequalities and theorems.

## Results

We illustrate the subtleties of fair sampling applied to the duality principle by considering an example where two internal degrees of freedom of a single physical system are coupled together and act as qubit and environment. We first consider the predictability and visibility measures of the qubit when the environment plays no role; the results are consistent with our conventional understanding of the duality principle. We then go on to show that an apparent violation of Eq. (1) can be obtained with conditional measurements.

Coupling to an environment can introduce a degree of decoherence to the initial system. This can be seen if one traces out the degree of freedom associated with the environment, leaving the initial system in a partially mixed state. In contrast, in our case, we select eigenstates of the environment, leaving the initial system in a pure state.

To keep the treatment simple, and without losing any power in our arguments (thanks to the possibility of employing a Schmidt decomposition on the joint state) we consider an environment that is also a qubit. A convenient way of realizing this situation in an optics framework is by using two eigenmodes of orbital angular momentum (OAM) of value 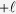 and 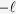, which we use as the qubit, and the polarization degree of freedom, which we use as the environment.

In our setup we produce the following state of OAM and polarization:


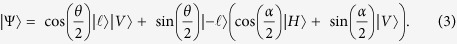


This state can be either separable or nonseparable; the degree of nonseparability can be controlled by the two angles *θ* and *α*. In particular, the state is nonseparable and maximally correlated for the configuration 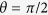 and *α* = 0. The density operator of the combined OAM and polarizaton qubits is given by 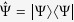. Therefore, the state of the OAM qubit ignoring polarization is given by the partial trace over the polarization states:


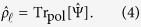


The visibility and predictability associated with the OAM qubit 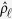 can be expressed in a compact way by using the Pauli operators in the OAM space applied in the following way^2^,^16^: 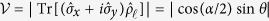 and 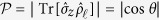. Taking the sum of the squares of these quantities, we find


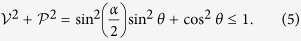


One can see that no values of *α* and *θ* lead to a violation of the duality principle, which is consistent with the duality relation of Eq. 1.

Now consider exploiting the correlation between the two qubits to obtain *conditional* values of 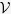 and 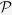. This idea has been previously explored theoretically by Bergou and Englert^14^. The state of the OAM qubit conditioned on successful postselection of the polarization degree of freedom is


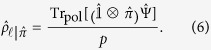


Here, 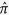 is a state of polarization, 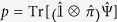 is the postselection probability, and the vertical bar notation means “given successful postselection on”. The visibility and the predictability of the OAM qubit that are conditioned on a successful postselection of the polarization qubit are


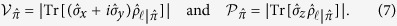


These two quantities satisfy the duality relation, Eq. 2, if the postselection used in both cases is the same. However, seemingly contradictory results can be obtained when measuring visibility and predictability conditioned on *different* postselections 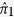 and 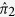. The most extreme case is when 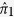 and 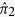 are orthogonal to each other. In this case one obtains


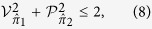


as 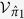 and 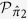 can independently reach 1. Such values can be observed even in experiments designed to measure visibility and predictability concurrently^11^.

In our experiment, the visibility and predictability that are obtained by postselecting the state 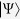 on vertical and horizontal polarizations (i.e. 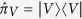 and 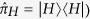 are


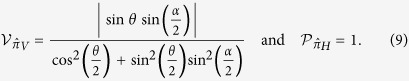


The sum of the squares of 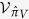 and 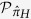 is always bounded below by 1 and above by 2.

This result is in apparent violation of the duality principle. The origin of this outcome is one of the main results of this paper: the apparent violation can only occur if the postselection states differ from each other. The orthogonality between our postselections, 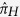 and 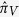, is a deliberate choice that produces the most extreme results, but an apparent violation can occur for other choices as well. From an inspection of the state (Eq. 3), we see that 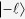 is the only OAM component associated with the horizontal degree of freedom. Therefore, a measurement of predictability conditioned on successful postselection of the horizontal polarization state will always equal to 1. Consider that this result combined with any measure of visibility that is greater than zero will result in an apparent violation of the duality principle. From the construction of our state, the only postselected polarization that will have zero visibility is the horizontal state. However, this is not the case if we consider the vertical postselection: in this case it is possible to achieve high-visibility fringes, albeit with an associated low probability. Combining the two results, each obtained with different postselections, gives rise to a sum of the squares of the visibility and predictability that is greater than 1.

One way to interpret this result is to associate a different qubit state for every state of the environment, i.e., the OAM qubit associated with horizontal polarization is different to that associated with vertical polarization. It is the postselection that alters the qubit. Consequently, one can measure visibility and predictability of each postselected qubit independently of the other. When the visibility and predictability of the OAM qubit vary from one state of the environment to the next, it is then easy to postselect a state with either high visibility or high predictability. In other words, states with high visibility and states with high predictability are both available.

The source of contradiction is specifically postselection: when there are many conditional measurements available, each with an associated probability of occurrence, the duality principle is satisfied if applied to the averaged visibility and predictability:


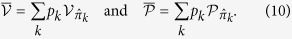


where in our case *k* labels vertical and horizontal polarizations. For the state 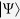, we have 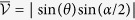 and 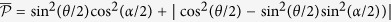, the sum of the squares of which is always bounded by 1. We see here that when postselection probabilities are correctly taken into account in addition to the outcomes of the conditional measurements, there is no violation of the duality principle. This result applies to any combined system and environment^14^.

The goal of the experiment is to perform the conditional measurements outlined in the previous section, highlighting the significance of coupling and postselection in tests of the duality principle. We first prepare a non-separable state of OAM and polarization; we then perform postselection of the polarization degree of freedom; and finally, we calculate the conditional visibility and predictability measures of the OAM degree of freedom.

**Figure 1:**
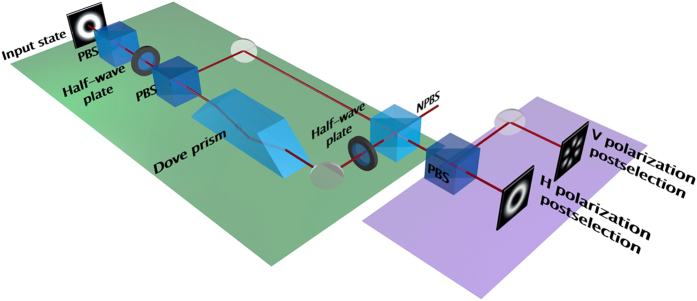
We first prepare a non-separable state of OAM and polarization (green area). We then perform postselection (purple area). The state of OAM is generated using a HeNe laser and a spatial light modulator (not shown). We control the amplitude in each path of the interferometer with a half-wave plate and a polarizing beam splitter (PBS) (this controls *θ*). Inside the lower path, a Dove prism reverses the handedness of the OAM mode and a second half-wave plate controls the polarization state inside one arm of the interferometer (this controls *α*). The non-polarizing beam splitter (NPBS) produces a superposition of the two paths, and the final PBS allows postselection on polarization. We measure the conditional visibilities and predictabilities for the *V* and the *H* outputs using images captured with a CCD camera.

The state of the system and environment is generated by inserting an OAM mode of 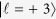 into a Mach-Zehnder interferometer with a Dove prism and half-wave plate in one of the arms; see Fig. 1. Before the interferometer, we use a collimated HeNe and a spatial light modulator to generate the OAM mode. The interferometer performs the role of “entangling” the OAM and polarization degrees of freedom, resulting in a non-separable state. The precise form of the joint OAM-polarization state is controlled by the two half-wave plates. The half-wave plate before the interferometer controls *θ* in the state; the half-wave plate inside the interferometer controls *α*.

A polarizing beam splitter after the output port of the interferometer projects onto the horizontal and vertical states of polarization. The horizontal polarization output is always composed of a single OAM mode, while the vertical polarization output is generally composed of two OAM modes of opposite handedness and with varying amplitudes, leading to a petal-shaped interference pattern.

**Figure 2:**
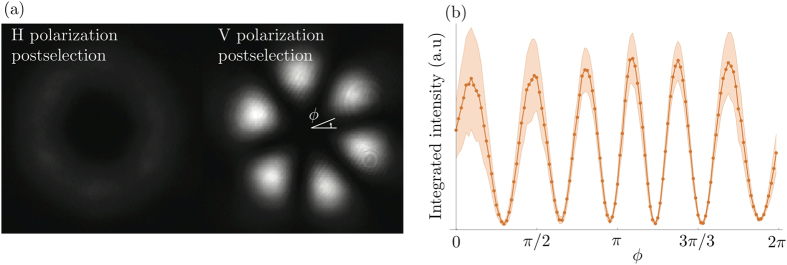
(**a**) A typical image of the *V* and the *H* postselected outputs captured with the CCD camera. The left part of the image (horizontal postselection) shows a very faint 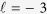 mode due to the low postselection probability, and there is a high predictability 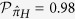 associated with this outcome. The right part of the image (vertical postselection) shows the intensity of a superposition of 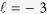 and 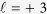 modes, and there are high visibility fringes 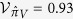 associated with this outcome. The value of 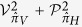 is equal to 1.83. (**b**) The azimuthally-integrated intensity of the vertical postselection shown in (**a**). Each data point corresponds to the average intensity in a 3° angular window. The shaded region indicates the error band, which is at one *σ*.

We use a CCD camera to record intensities of the modes and then calculate the visibility and distinguishability measures. Figure 2 shows a typical image captured by the camera. In order to obtain an apparent violation of the duality relation in the way described above, we measure the predictability after postselection of horizontal polarization and the visibility after postselection of vertical polarization. We measure 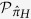 as the difference in intensity of the two arms of the interferometer. Finally, we measure 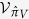 with respect to the vertical polarization from the plot that we obtain by integrating radially with respect to the centre.

## Discussion

**Figure 3:**
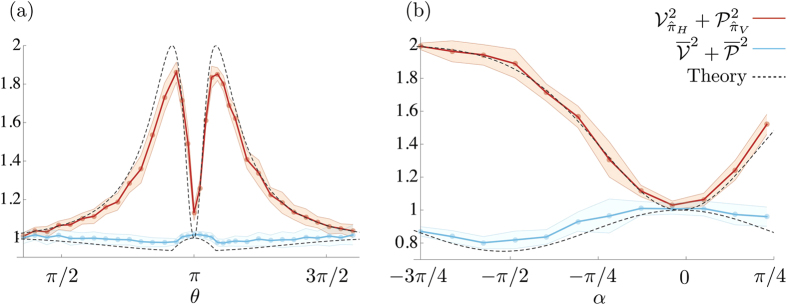
(**a**) Duality relation as a function of the initial polarization for a weak environment coupling 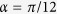. (**b**) Duality relation as a function of coupling for a fixed initial polarization 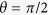. The red lines show that it is possible obtain values of 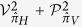 higher than 1 for a large range of states and couplings. The blue lines show that when the average quantities are used, the sum is never greater than 1. For the plots given above, the error band is at one *σ*.

Our experimental results are shown in Fig. 3(a,b). The data show the sum of the squares of the measured conditional visibilities and predictabilities together with the average quantities. Figure 3(a) shows data for a range of values of *θ*; recall that this controls the polarization state before the interferometer. Figure 3(b) shows data for a range of values of *α*; this controls the polarization state of the lower path of the interferometer. In both figures, we see that the sum of the squares of the conditional measurements exceeds 1 (see the red curves). In contrast, the sum of the squares of the averaged quantities never exceeds 1 (see the blue curves).

Consider the result in Fig. 3(a), where the highest value of 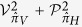 appears when *θ* = *π* ± *α*, where *α* = *π*/12 in the example is a small angle. This state corresponds to when the input polarization state is close to, but not quite, horizontal. In this case, almost all the light enters the lower arm of the interferometer, but due to the small component of vertical polarization of the input state, there will be a small component in the upper arm. The small amplitude of the vertical polarization state in the upper arm can be matched in the lower arm by rotating the wave plate (*α*). The light that exits the interferometer now has a large horizontal component that only passed through the lower arm and a small vertical component that has passed through both arms. A measurement of the predictability conditioned on the horizontal polarization state will be equal to unity, and a measurement of the visibility conditioned on the vertical polarization state will also be equal to unity. Under these conditions, we report the observation of both high visibility and high predictability without violating the principle of duality.

We can summarize our result in a simple way by considering two two-level systems that are coupled, or entangled, with each other. As stated previously, the coupling between the two systems means that postselection of one system creates a new state in the other. It follows that two different postselections of the first will create two different states for the second. It is at this point that there is potential for a fair-sampling criterion to no longer be satisfied. The fair-sampling criterion in the context of this work would be that the detected states used to collect and report data are representative of the system of interest. However, we see here that the two different postselections provide a potential mechanism to use two different states for the visibility and which-way measurements, and as they are not representative of the system of interest, they violate this criterion.

Our work gives experimental evidence of some of the consequences of not respecting the fair sampling condition. We show that if a qubit is coupled to its environment, it becomes possible to obtain simultaneous high values for conditional measures of visibility and predictability. In this case the predictability and visibility values can lead to an apparent violation of the duality principle. To achieve such a result, we are required to disregard certain measurement outcomes, enforcing a fair-sampling-like loophole via postselection.

We note that although our experimental procedure allowed us to purposely obtain simultaneous high values which lead to an obvious violation of an algebraic bound, there can be realistic experimental cases where an inadvertent postselection could be performed without necessarily obtaining a clear violation. In these cases, detecting the loophole might be much more difficult. We note that under no circumstance do we claim that a violation of the duality principle is possible. Rather, we seek to highlight certain experimental conditions where apparent violations occur. As such, this work demonstrates the crucial role of fair-sampling in tests of the duality principle and potentially in other fundamental tests of quantum mechanics.

## Additional Information

**How to cite this article**: Leach, J. *et al.* The duality principle in the presence of postselection. *Sci. Rep.* **6**, 19944; doi: 10.1038/srep19944 (2016).
